# Spondylocostal dysostosis with split cord malformation in a resource-limited setting: A case report

**DOI:** 10.1016/j.radcr.2025.06.013

**Published:** 2025-06-27

**Authors:** Kidus Demelash, Suleiman Belay, Haymanot Alebachew, Gebreegziabher Hailay, Fire Yishar, Abate Weldetsadik

**Affiliations:** a School of Medicine, Saint Paul Hospital Millennium Medical College, Addis Ababa, Ethiopia; b School of Medicine, College of Medicine and Health Sciences, University of Gondar, Gondar, Ethiopia; c Department of Radiology, Saint Paul Hospital Millennium Medical College, Addis Ababa, Ethiopia; d Department of Pediatrics and Child Health, Saint Paul Hospital Millennium Medical College, Addis Ababa, Ethiopia

**Keywords:** Spondylocostal dysostosis, split cord malformation, Congenital anomaly, Case report

## Abstract

Spondylocostal dysostosis (SCD) is a rare congenital disorder marked by severe vertebral and rib malformations, often resulting in thoracic insufficiency and respiratory issues. This report presents an 11-day-old neonate with persistent respiratory distress and chest wall deformities**,** found to have multiple rib agenesis, vertebral segmentation defects, and an associated split cord malformation (SCM). Although early surgical intervention can improve outcomes, the family chose conservative management due to accessibility and socioeconomic factors. The case highlights the complexities of managing Spondylocostal dysostosis (SCD), particularly when complicated by split cord malformation (SCM), and emphasizes the need for early diagnosis, comprehensive imaging, and individualized treatment strategies to optimize prognosis.

## Introduction

Spondylocostal dysostosis (SCD) is a rare congenital skeletal disorder first described by Jarcho and Levin in 1938, characterized by thoracic insufficiency resulting from complex vertebral and rib anomalies [1]. The condition is marked by multiple congenital malformations, including abnormal segmentation of the spine and ribs, short neck and trunk, and normally proportioned limbs. Rib anomalies may include absent, fused, or overgrown ribs, while vertebral defects commonly involve fusion, hemivertebrae, or block vertebrae [2].

Although the precise prevalence of SCD remains uncertain, it is widely recognized as an exceedingly rare disorder [3]. In this report, we present the clinical features and imaging findings of an 11-day-old male neonate diagnosed with SCD associated with split cord malformation (SCM), who presented with respiratory distress from birth.

## Case presentation

An 11-day-old male neonate was referred to our tertiary care facility with persistent respiratory distress noted since birth. He was delivered at home at full term via spontaneous vaginal delivery, weighing 3.8 kg. The APGAR score at birth was not documented. Within the first few hours of life, the infant exhibited rapid breathing and noticeable chest retractions. He was subsequently admitted to the Neonatal Intensive Care Unit (NICU) at a local hospital, where he received intranasal oxygen and empirical antibiotics. He remained in the NICU for 8 days with a provisional diagnosis of early-onset neonatal sepsis before being referred for further evaluation and specialized care.

The neonate was born to a gravida III para III mother, who had been diagnosed with type 2 diabetes mellitus 4 months prior to conception. She had been on a regimen of insulin (10 IU in the morning and 20 IU in the evening) for one year, with documented good glycemic control throughout the pregnancy. The mother discovered she was pregnant during the second trimester and initiated antenatal care (ANC) at that time. Her care included daily supplementation with iron, 400 micrograms (mcg) of folic acid, and calcium, all starting in the second trimester. She also received 2 doses of tetanus toxoid in accordance with standard prenatal guidelines. An obstetric ultrasound conducted at the local health center during the pregnancy was unremarkable. There was no significant family history of congenital anomalies or consanguinity. The mother denied any exposure to teratogenic drugs, fever, urinary incontinence, or other chronic conditions during pregnancy. Both the father and the patient’s 2 siblings were reportedly healthy and without congenital anomalies.

**Fig. 1:**
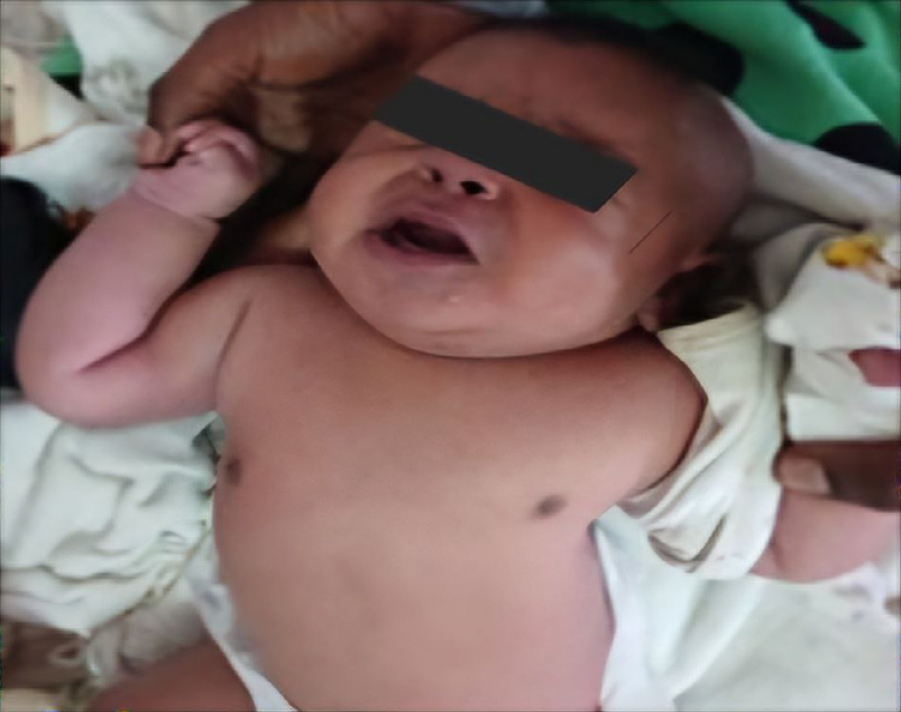
Clinical picture of the neonate demonstrating an asymmetrical chest wall. Subcostal and intercostal retractions are evident with each breath.

On examination, the infant displayed signs of respiratory distress with a heart rate of 180 bpm, respiratory rate of 88 breaths per minute, and an axillary temperature of 36.6°C. Oxygen saturation levels ranged between 78%-82% on room air and improved to 93% with inhaled oxygen. The thoracic cage was visibly asymmetrical with diminished bilateral chest expansion (Fig. 1). Notably, heart sounds were auscultated on the right side of the chest. The left rib cage was underdeveloped, accompanied by mild subcostal and intercostal retractions during respiration. On auscultation air entry was comparable on both sides. No additional abnormalities were identified on physical examination of other systems.

**Fig. 2:**
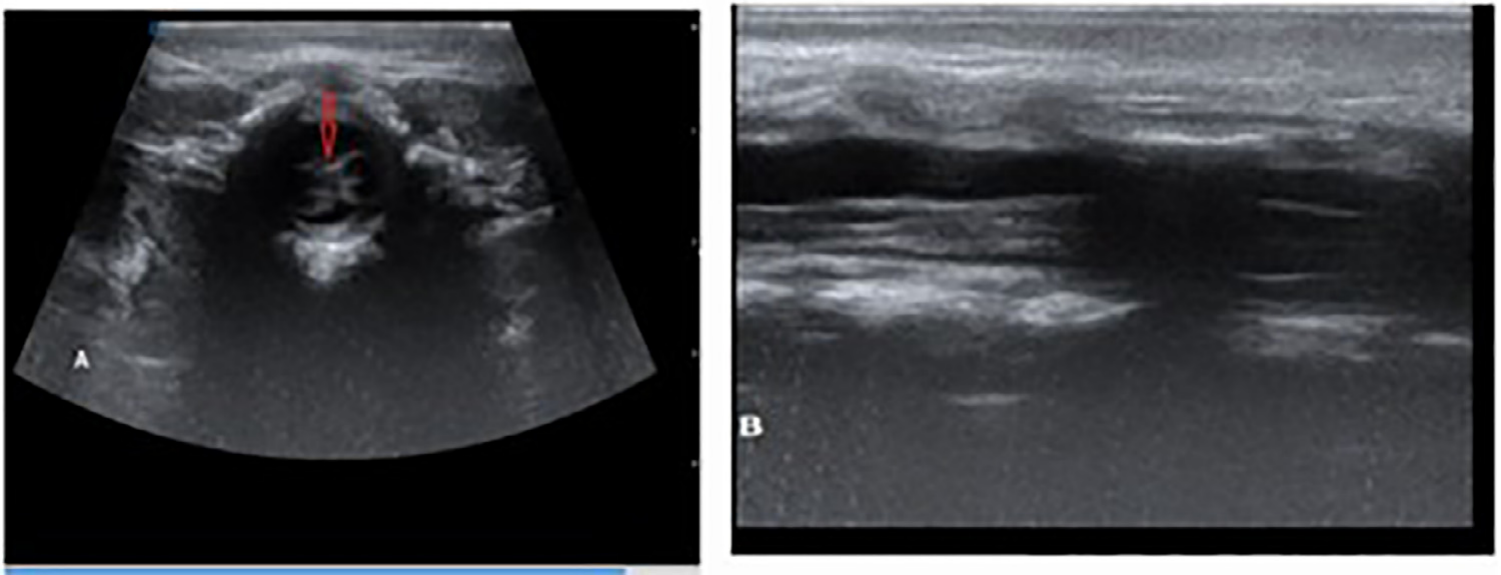
Anteroposterior (AP) radiograph of the thoracic spine showing mild levoscoliosis in the mid-thoracic region. Vertebral anomalies include formation and segmentation defects between T6 and T8 (yellow arrow). The left 5th, 6th, and 7th ribs are completely absent (red star). Partial fusion is noted between the 11th and 12th ribs (yellow star), and the left 9th rib appears bifid (green star). The heart is shifted to the right side (dextroposition) (black arrow).

An anteroposterior (AP) chest and spinal radiograph revealed multiple rib and vertebral anomalies along with thoracic dextroscoliosis. There was hypoplasia of the left 1st, 4th, and 7th ribs, and complete agenesis of the 5th and 6th ribs. Fusion was observed among the lower left ribs from the 8th to 12th segments. Several thoracic vertebrae displayed segmentation defects, including hemivertebrae. The heart was dextropositioned. The findings were consistent with SCD (Fig. 2).

**Fig. 3:**
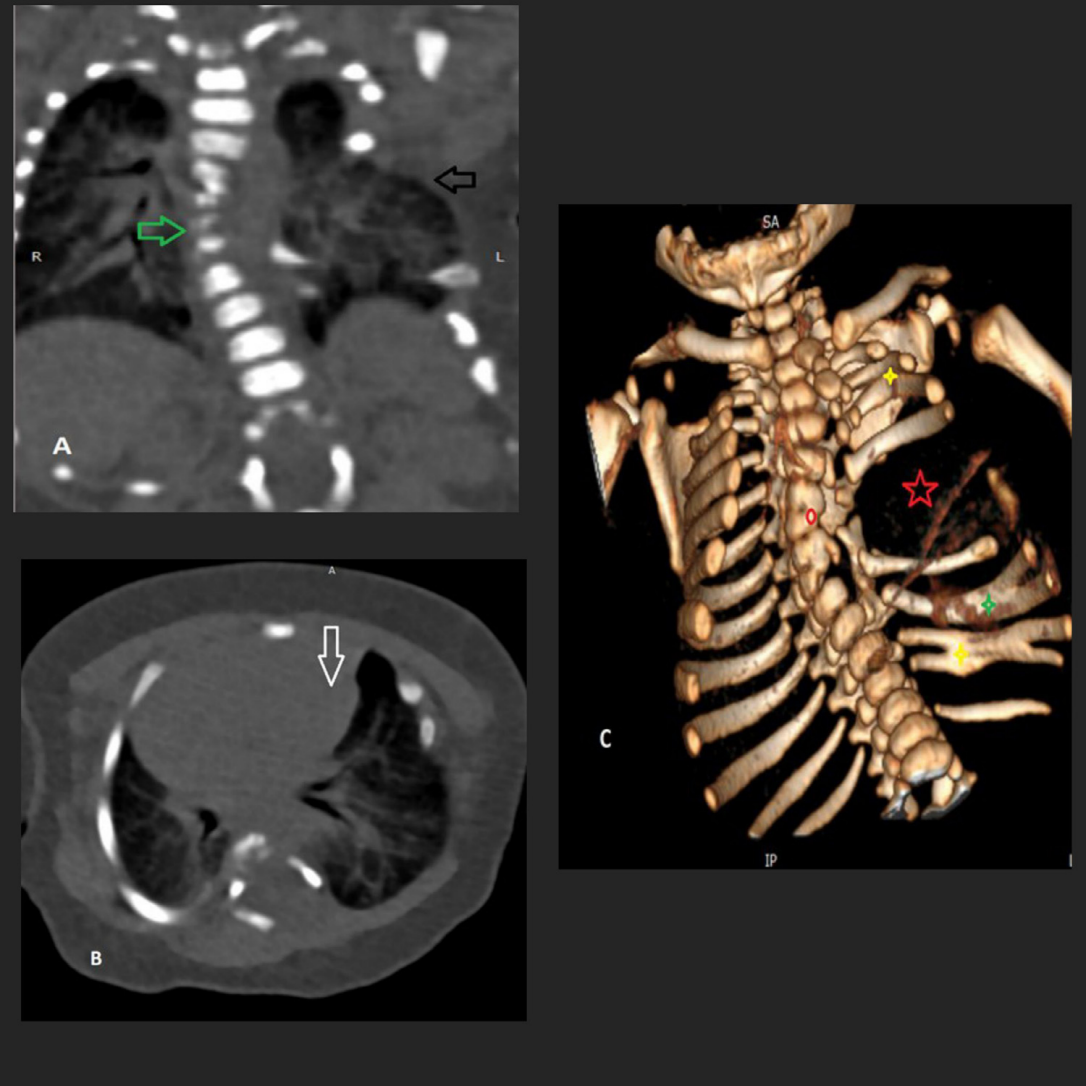
Chest CT scan findings. (A) Coronal view, (B) axial view, and (C) 3D reconstruction confirm absence of the left 5th, 6th, and 7th ribs (red star). Partial fusion is observed in the mid-segments of the 2nd and 3rd ribs and the 10th and 11th ribs (yellow star). The left 9th rib appears bifid (green star). The heart is dextropositioned (white arrow), and herniation of the left lung through the rib defect is evident (black arrow). A left-sided vertebral bar at the T7-T8 level is noted, as well as a hypoplastic butterfly vertebra at T6. Mild levoscoliosis is present in the mid-thoracic spine (red circle).

A subsequent computed tomography (CT) scan provided further detail, confirming thoracic dextroscoliosis, hemivertebrae, and absent left ribs (5th-7th), as well as fusion and bifurcation of other ribs. Additionally, a left-sided vertebral bar at T7-T8 and a hypoplastic butterfly vertebra at T6 were noted. Dextroposition of the heart was evident, along with herniation of the left lung through the rib cage defect (Fig. 3). On echocardiography, no cardiac structural anomalies were identified.

To assess for further congenital anomalies, transfontanelle and abdominal ultrasonography were performed, both yielding unremarkable results. However, a lumbosacral ultrasound revealed an anechoic cystic structure at the thoracocervical junction exerting posterior pressure on the spinal cord. There was also evidence of cord splitting from T12 to L2, terminating at L2-L3, consistent with diastematomyelia (SCM) (Fig. 3). An MRI was recommended for detailed spinal assessment, but the family was unable to afford the imaging.

The diagnosis of SCD with associated SCM and thoracic insufficiency syndrome was established based on clinical presentation and imaging findings. Multidisciplinary consultation was sought involving neonatology, pediatric orthopedics, and pediatric neurosurgery.

Given the thoracic deformities contributing to respiratory compromise, surgical intervention such as rib expansion using vertical expandable prosthetic titanium ribs (VEPTR) was advised to improve thoracic volume and lung function. Additionally, surgical correction of the spinal malformation was discussed as a future consideration to prevent neurological deterioration.

Supportive care was initiated, including continued oxygen supplementation, nutritional support via orogastric feeding, and close monitoring of respiratory status. Parents received comprehensive counseling regarding the child’s condition, prognosis, and potential benefits and risks of surgical intervention.

Despite detailed explanations and reassurance, the family declined surgical management and chose to take the infant home against medical advice.

## Discussion

Jarcho-Levin syndrome (JLS), also referred to as SCD, is an umbrella term for a rare congenital skeletal disorder primarily affecting the vertebral column and ribs. The condition typically presents with a short neck and trunk, normal-sized limbs, and multiple vertebral anomalies at various levels of the spine, often accompanied by costal defects [3]. The morphological characteristics observed in the neonate presented in this case are consistent with the typical clinical features of JLS.

Globally, fewer than 400 cases of JLS have been reported, with only about 15 cases documented in the Indian medical literature [3]. The syndrome encompasses 2 genetically distinct entities: spondylocostal dysostosis (SCD) and spondylothoracic dysplasia (STD). Both disorders involve multiple vertebral and rib anomalies but can be differentiated radiographically. In SCD, the ribs are frequently asymmetrically malformed—being fused, absent, or hypertrophied—while in STD, the ribs are structurally normal but symmetrically fused at the costovertebral joints, resulting in a characteristic “crab-like” or “fan-shaped” thoracic appearance [4].

SCD is associated with a high rate of neonatal morbidity and mortality, predominantly due to thoracic insufficiency syndrome, which compromises pulmonary function and respiratory mechanics [5]. Over the years, advances in genetic research, imaging modalities, and case-based studies have significantly improved the understanding of this condition. Clinically, SCD often manifests with a short-webbed neck, short trunk, protuberant abdomen, and varying degrees of scoliosis. The condition may also be accompanied by other congenital anomalies including urogenital and anorectal malformations, hernias, cardiac defects, neural tube defects, and limb deformities. Radiologically, SCD is characterized by a spectrum of vertebral segmentation anomalies such as hemivertebrae, butterfly vertebrae, absent vertebrae, and block vertebrae. The disorder may be inherited in either an autosomal dominant or autosomal recessive pattern [6].

The association of SCD with neural tube defects is well documented in the literature. It is hypothesized that the vertebral abnormalities arise from a developmental disturbance during the 4th to 8th weeks of gestation, a critical period when multiple chondrification centers form around the notochord to create a complete cartilaginous model of the vertebrae. Disruption in the formation or fusion of these centers can result in hemivertebrae or butterfly vertebrae, with secondary rib anomalies arising from the underlying vertebral malformations [7]. A case report from Karnataka, India, for instance, described a neonate with associated anomalies such as hydrocephalus, hydroureteronephrosis, and meningomyelocele; however, such anomalies were not observed in the neonate presented in our case [3].

Management of SCD is both medical and surgical. Initial efforts are directed toward the stabilization of respiratory function and prevention of recurrent pulmonary infections. Surgical interventions are tailored based on the severity of thoracic insufficiency, the extent of chest wall defects, and the presence of associated complications. Among the most effective surgical options are Vertical Expandable Prosthetic Titanium Ribs (VEPTR), which are used to expand the thoracic cavity and improve lung function. Chest wall reconstruction using latissimus dorsi flap transfers has also been described for managing structural deformities [2].

Split cord malformation (SCM), when present, typically requires neurosurgical correction to prevent progressive neurological impairment. Surgical management of SCM involves decompression, removal of bony or fibrous septations, and dural repair [8]. Although surgical correction was strongly advised due to the severity of thoracic anomalies and risk of further respiratory compromise, the parents opted against surgical intervention and chose to discharge the infant against medical advice. This underscores the importance of parental counseling, socioeconomic considerations, and early multidisciplinary involvement in the management of complex congenital anomalies such as SCD.

## Conclusion

SCD is a rare congenital disorder characterized by vertebral and rib anomalies, often leading to thoracic insufficiency and associated malformations such as SCM. Early diagnosis and multidisciplinary management are crucial for optimizing clinical outcomes. Surgical interventions can play a key role in improving respiratory function and preventing complications.

## Patient consent

Written informed consent for the publication of this case report and any accompanying images was obtained from both legal guardians of the patient.
